# Odor cueing during sleep improves consolidation of a history lesson in a school setting

**DOI:** 10.1038/s41598-022-14588-x

**Published:** 2022-06-20

**Authors:** Vanessa Vidal, Alejo R. Barbuzza, Leonela M. Tassone, Luis I. Brusco, Fabricio M. Ballarini, Cecilia Forcato

**Affiliations:** 1 Laboratorio de Sueño y Memoria, Departamento de Ciencias de la Vida, Instituto Tecnológico de Buenos Aires (ITBA), Capital Federal, Buenos Aires Argentina; 2 Consejo Nacional de Investigaciones Científicas y Tecnológicas (CONICET), Capital Federal, Buenos Aires Argentina; 3 Instituto de Biología Celular y Neurociencias “Prof. E. De Robertis” (IBCN), Facultad de Medicina, Universidad de Buenos Aires (UBA)-Consejo Nacional de Investigaciones Científicas y Técnicas (CONICET), Capital Federal, Buenos Aires Argentina; 4 Centro de Neuropsiquiatría y Neurología de la Conducta (CENECON), Facultad de Medicina, Universidad de Buenos Aires (UBA), Capital Federal, Buenos Aires Argentina; 5 Departamento de Ciencias de la Vida, Instituto Tecnológico de Buenos Aires (ITBA), Capital Federal, Buenos Aires Argentina

**Keywords:** Neuroscience, Cognitive neuroscience, Learning and memory

## Abstract

Sleep is a key factor in memory consolidation. During sleep, information is reactivated, transferred, and redistributed to neocortical areas, thus favoring memory consolidation and integration. Although these reactivations occur spontaneously, they can also be induced using external cues, such as sound or odor cues, linked to the acquired information. Hence, targeted memory reactivation during sleep represents an advantageous tool for improving memory consolidation in real-life settings. In this study, our goal was to improve the consolidation of complex information such as that of a history lesson, using a school study session in the presence of an odor, and a reactivation round while sleeping at home on the same night of the acquisition, without using additional study sessions. We found that complex information can be associated with an odor in the classroom and that one session of reactivation during the first night of sleep in the students’ houses improves its consolidation. These results bring new evidence for the implementation of reactivation during sleep in real-life settings.

## Introduction

Memories are not immediately fixed after acquisition. On the contrary, at first they are labile, and then they are followed by a stabilization process known as consolidation, where the memory trace becomes protected against interference^1^. Sleep plays a principal role in memory consolidation. During sleep, recently acquired information is spontaneously reactivated, transferred, and redistributed to neocortical stores, favoring memory consolidation and integration^2^. However, it is important to highlight that not all memories are equally benefited during sleep. Emotionally salient stimuli, rewarded information, and intention and instruction to remember (all the information that involves future relevance) are the ones particularly benefited by sleep^3,4^, but see Davidson et al. (2021)^5^.

In recent years, sleep has been proposed as a tool to improve education^6,7^. Preliminary results showed that sleeping after learning enhances subsequent retrieval also in school settings^8–10^. However, we should take into account that not necessarily all the information acquired at school is taken as relevant by our brain and, thus, it can easily be forgotten. Nevertheless, there are plenty of studies showing that when a cue (e.g. odor or sound) previously linked to a task (e.g. word pairs) is presented again during sleep, particularly during the first Non-Rapid Eye Movement (NREM) sleep cycles, memory strengthening and/or protection against interferences emerges as a result of the induced reactivation^3,11–14^. Thus, the presentation of specific cues during learning and subsequent sleep could guide which content would be consolidated in the sleeping brain (e.g. a school history lesson), independently of its relevance. In this way, targeted memory reactivation during sleep becomes a promising tool to improve memory consolidation in real-life settings.

Recently, Neumann et al. (2020) published the first study of memory reactivation during sleep involving vocabulary learning in a regular school setting. Participants learned different sets of German-English vocabulary pairs presented at school without an odor cue^15^. Then, they went through four different conditions of odor presentation: 1) during studying time at home and during sleeping at night for 7 days, and during the vocabulary test on day 7; 2) during studying time at home and during sleeping at night for 7 days; 3) during studying time at home only; 4) no presentation of the odor. Interestingly, the researchers found that the odor reactivation during the whole night without sleep monitoring improved memory retention as in controlled experiments in the lab only if the odor had been previously presented during the learning sessions.

**Figure 1:**
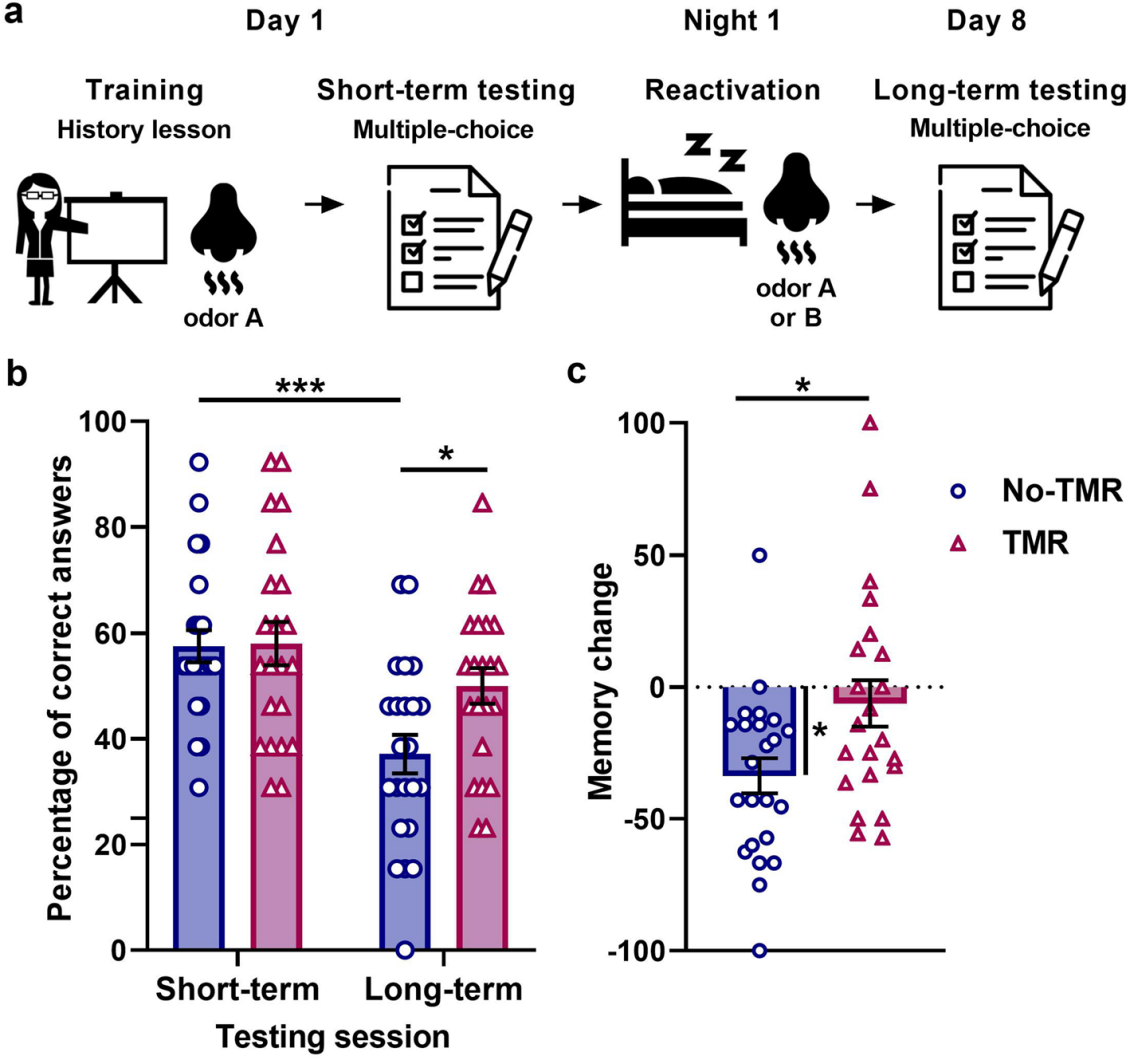
(**a**) The history lesson was presented by their teacher, using a PowerPoint presentation on a projector screen. Each slide was shown for 1.5 min as she commented on them orally (day 1). Short-term testing: Students made an evaluation consisting of 13 multiple-choice questions with four possible answers and one correct answer; they had no time limit to answer them and all the right answers had been previously presented on the slides during the training. Night 1: Reactivation: At home, half of the volunteers used dispensers containing the congruent odor A (coconut), whereas the other half used dispensers containing the incongruent odor B (violets) (depending on which group they were assigned to) at the time they went to sleep for 1.5 h. The odor was sprayed every 15 min. Day 8: Long-term testing: Students made another evaluation consisting of 13 multiple-choice questions with four possible answers and one correct answer; they had no time limit to answer them and all the right answers had been previously presented on the slides during the training on day 1. (**b**) Mean percentage of correct answers at testing sessions on days 1 and 8 ± SEM for the TMR and No-TMR groups. (**c**) Mean memory change ± SEM for the TMR and No-TMR groups. *, p < 0.05; ***, p < 0.001. Icons made by Freepik.

Here, we aimed to study the effect of cueing a more complex school curriculum content, by presenting an odor cue to sixth-year high-school students while learning a history lesson at school, and by reactivating the memory during the first night of sleep after acquisition, in their homes. To this end, we performed a two-day experiment (Fig. 1). On day 1, the students received a history lesson about the city of Petra and the Nabatean culture and commerce by their teacher of Political Economics in the presence of a coconut odor and were then immediately evaluated to measure the initial level of learning (short-term testing). Then, during the first hour and a half of sleep at home, half of the students received the same odor (coconut) (self-administered) (Targeted Memory Reactivation, TRM, group), while the other half received violets odor (control condition, No Targeted Memory Reactivation, No-TMR, group). Finally, seven days later, i.e. on day 8, students were tested again in the absence of the odor (long-term testing) (Fig. 1a).

## Results

To study the effect of odor cueing during sleep on memory consolidation in a school setting, we conducted a two-day experiment (Fig. 1). On day 1, the students received a history lesson provided by their teacher in the presence of an odor (coconut) and were immediately evaluated (short-term testing). Half of the participants received the same odor (coconut, Targeted Memory Reactivation group, TMR) or a control odor (violets, No Targeted Memory Reactivation group, No-TMR), during the first night of sleep. They were finally evaluated on day 8, (long-term testing) (Fig. 1a).

### Odor cues during sleep improved memory retention

Repeated measures ANOVA revealed a “group” per “session” interaction (F(1,43) = 4.76, *p* = 0.035, η^2^_p_ = 0.1). Thus, we performed simple effects analyses of “group” within each level of “session”. We found that, on day 1, the groups reached a similar percentage of correct answers (Fig. 1b, TMR group: 58.0 ± 4.1; No-TMR group: 57.5 ± 3.1, simple effects F(1,43) = 0.010, *p* = 0.919), whereas on day 8, the TMR group showed a significantly higher percentage of correct answers than the No-TMR group (Fig. 1b, TRM group: 50.0 ± 3.4; No-TMR group: 37.1 ± 3.6, simple effects F(1,43) = 6.68, *p* = 0.013, η^2^_p_ = 0.1). Simple effects analysis of “session” within each level of “group” showed that there was a significant memory decay between the short-term and long-term memory sessions for the No-TMR group and a trend for the TMR group (Fig. 1c, simple effects, F_No-TMR_(1,43) = 26.54, *p* < 0.001, η^2^_p_ = 0.4 ; F_TMR_(1,43) = 3.94, p = 0.053, η^2^_p_ = 0.08).

Additionally, we found that the TMR group had a significantly lower memory change (defined as the percentage of correct answers achieved on day 8 with respect to day 1, see section "Methods, Statistical Analysis") than the No-TMR group (Fig. 1c, TMR group: -6.2 ± 8.8, No-TMR group: − 33.7 ± 6.6; Two-sample t-test t(43) = − 2.51, *p* < 0.016, Cohen’s d = 0.75). Furthermore, the memory change of the No-TMR group significantly differed from zero (One-sample t-test(22) = − 5.08, *p* < 0.001, Cohen’s d = − 1.06), whereas the memory change of the TMR group did not differ from zero (One-sample t-test(21) = − 0.71, *p* = 0.485).

### Control measures

There were no significant differences between groups for the state anxiety on day 1 (TMR group: 33.6 ± 1.3; No-TMR group: 36.6 ± 2.1, F(1,40) = 1.391, p = 0.245), the state anxiety on day 8 (TMR group: 34.8 ± 2.0; No-TMR group: 34.1 ± 2.0, F(1,35) = 0.048, p = 0.828), the trait anxiety (TMR group: 39.0 ± 1.9; No-TMR group: 39.8 ± 2.8, F(1,34) = 0.054, p = 0.818), and the Pittsburgh sleep quality index (TMR group: 5.4 ± 0.4; No-TMR group: 5.8 ± 0.8, F(1,34) = 0.191, p = 0.665).

## Discussion and conclusion

Here, we showed that targeted memory reactivation during sleep improves memory consolidation of complex information such as that of a history lesson in a school setting. We also showed that one session of cueing during sleep during the first night after the lesson is sufficient to obtain a significant effect on memory consolidation. In comparison to that observed by Neumann et al. (2020), who applied the cue during seven consecutive nights, we observed a significant effect with only one night of reactivation^15^. However, it remains to be evaluated whether the benefit of additional rounds of reactivation is greater than just one reactivation.

Another difference between the study by Neumann et al. (2020) and our study is that we presented the odor at school during the class, whereas Neumann et al. presented it during individual study sessions at volunteers’ houses^15^. This result demonstrates the successful association between the odor and the lesson in the school setting, allowing subsequent reactivation at home during sleep, without having to undergo additional sessions of study outside the classroom. Nevertheless, the disadvantage of presenting the odor cue in the classroom is the risk that if the classroom is not properly depleted of the odor after the lesson, the cue could potentially end up associated with other information. Furthermore, it is important to highlight that, between memory acquisition on day 1 and evaluation on day 8, the TMR group showed a non-significant decrease of 6.2% in their performance, while the No-TMR group had a significant decrease of 33.7%. This would mean that the information learned at school could be retained for at least one week if it is reactivated during sleep by presenting cues associated with the learned material. Related to this, by using classical music to reactivate a college Microeconomics lecture during sleep, Gao et al. (2021) showed that targeted memory reactivation increased the likelihood of passing the test relative to the control condition (being ≥ 70 the cutoff for passing a test)^16^. In our study, students reached a lower level of performance in both the short- and long-testing sessions. This difference could be due to the distinct nature of the lecture or to differences in motivation^17^. Regarding the former, in Gao et al. (2021), the lecture was about microeconomics, a subject related to previous knowledge of mathematics, whereas, in our study, students learned a history lesson about the city of Petra, which was unknown declarative information for the students. Regarding motivation, students go to university because of their own initiative, whereas, in Latin-American countries, going to high school is compulsory, making motivation a distinctive factor that could be changing memory codification between studies. However, it is important to take into account that, for real exams at school, students usually study at home, whereas here they only had their teacher’s lesson and did not study further at home. In terms of school grades, adding further rounds of study at home in combination with odor reactivation would increase the students’ performance.

Importantly, reactivation studies have shown that cues presented during Slow Wave Sleep (SWS) benefit declarative memory consolidation, whereas those presented during REM have no effect^11^. Thus, considering that the first 40 min of sleep (first sleep cycle) are rich in SWS, reactivation could be restricted to the first part of sleep, with no need for the subject to be exposed to the odor all night. Furthermore, although odor cues do not interfere with sleep structure as much as sound cues do^18^, they can modify sleep architecture^19,20^. Hence restricting subjects' exposure to the odor is desirable.

Neumann et al. (2020) found a tendency to larger memory effects of the cue presentation during testing^15^. Several studies have shown that maintaining the enriched context during testing favors memory retrieval^21^. For example, Forcato et al. (2007) showed that learning five pairs of nonsense syllables in an enriched environment formed by a color light, an image, and music improved retrieval of the pairs when the enriched context was presented again during the testing session three days later, compared to a control group (without color light, image or music)^21^. This aspect should also be taken into account at the time of evaluation at school to obtain better performance.

Transferring a laboratory experiment into a real-life situation is challenging and presents constraints that would not be present in lab conditions. For example, one limitation of our study was the low sample size. However, this allowed us to have the same teacher in both courses, lowering this way the variability between them. Moreover, since data from the two courses were collected at the same time during the COVID-19 pandemic, increasing the N size would have implied performing additional experiments and thus the introduction of a new variable, i.e. a new stage in the pandemic, which could have potentially introduced noise in our data.

One important point to take into account is whether targeted memory reactivation increases the capacity limit of the sleep effect on memory consolidation, or biases consolidation to specific information^22,23^. In this context, it may be debated whether targeted memory reactivation is detrimental to uncued memories^24^. If the negative effect for uncued memories occurs in school settings, it would be prejudicial for other material learned at school that has not been cued during sleep. One possible solution to this potential drawback is to apply targeted memory reactivation in combination with other strategies able to improve sleep-dependent memory processing. For example, Feld and Diekelmann (2020) proposed that several sleep-related interventions, such as applying sleep hygiene strategies, would improve the quality of sleep^24^ and consequently memory consolidation of information with future relevance^4^. It is important to highlight that it has recently been suggested that sleep favors all kinds of memories independently of their relevance^5^, adding further importance to sleep-related strategies. Other interventions proposed by Feld and Diekelmann (2020) are transcranial electrical stimulation, closed-loop auditory stimulation, and hypnosis, all of which pointed to improving SWS^24^. Other interventions such as the use of a novelty near memory acquisition^25^, successive reminder presentation of a previously learned task^26^, or acute stress induction^27^ could be used to improve the uncued information.

Another limitation of our study is the fact that we did not cross the odor types between groups (i.e. TMR group with violet odor, and No-TMR group with coconut odor). To address the possibility that the memory improvement was due to nonspecific effects of the chosen odor on sleep, we decided to conduct the experiment with an odor different from the one used by Rasch et al. (2007) to study targeted memory reactivation, as well as different from that used by Neumann et al. (2020); in both cases, the authors used a rose odor^11,15^. Thus, the fact that memory improvement is observed using different odors (i.e. coconut and roses) to reactivate memories during sleep indicates that odors in general can be used to promote targeted memory reactivation. Furthermore, in the present study, the students did not know whether the odor they were going to use in their houses while sleeping was the same as the odor present in the history lesson or not. However, as the reactivation started 15 min after the lights turned off, if sleeping took longer they could have identified the odor adding further limitations to the study.

In conclusion, our present results not only support previous data showing that targeted memory reactivations can be implemented in real-life settings^14^ but also showed that complex information such as that of a history lesson can be associated with an odor in the classroom and that its consolidation can be improved with just one session of reactivation during the first night of sleep in the students’ homes.

## Methods

### Participants

A total of 68 healthy 17 to 18-year-old high-school students (27 females and 41 males) from two different 6th-year courses from the same school (who shared the same teacher) volunteered for the study. None of the participants reported having any history of neuropsychiatric disorders, use of drugs, or being sick or taking any medication during the experiment. Their parents or legal guardians signed a written informed consent approved by the Biomedical Research Ethics Committee of the Instituto Alberto C. Taquini (Buenos Aires, Argentina), previously to the students’ participation in the study.

Data from 23 participants were excluded from the study because they did not participate in the testing session on day 8 (16 of them) or did not match the learning criteria of 30% of correct responses on day 1 (7 of them). Thus, the final sample was 45 participants. Unlike that performed by Neumann et al. (2020), who presented the odor all night long^15^, our odor was presented for 1.5 h to guarantee the cueing to be present during the first cycle of NREM sleep^2^.

### Experimental design

The study was conducted on two different days, separated by a seven-day interval. Both the protocol and the consent were approved by the Biomedical Research Ethics Committee of Instituto Alberto C. Taquini (Buenos Aires, Argentina), in accordance with the principles expressed in the Declaration of Helsinki. Two days before the beginning of the experiment, students were told that they could volunteer to be a part of a study about learning and memory and that they would be able to know the hypothesis at the end of the study. On day 1, the classrooms were odorized using spray dispensers before the students entered the room.

Both courses received a history lesson (training session) of 20 min provided by the same teacher of Political Economics, in the presence of coconut odor. To perfume the classroom, we used one spray dispenser for every three students. The history lesson was about the city of Petra and the Nabatean culture and commerce. When the teacher finished the lesson, the spray dispensers were turned off. Afterward, the students made a multiple-choice exam of 13 questions about the lecture (short-term testing session). After the exam, they completed the State-Trait Anxiety Inventory (STAI)^28^ and the Pittsburgh Sleep Quality (PSQ) Index^29^.

In each classroom, students were randomly assigned to the Targeted Memory Reactivation (TMR) or the No-Targeted Memory Reactivation groups (No-TMR), and each was provided with an automatic spray dispenser according to the group they were assigned to: the TMR group was given the same odor that was presented in the classroom during the lesson (coconut), and the No-TMR group was provided with a different odor (violets). Students were instructed to use the spray dispensers in their homes the night of the first experimental day (reactivation session). They were told to set the initiation time of the dispenser at the time they went to sleep and the finishing time at 1.5 h later. They returned the dispensers the following day. On day 8, they made another multiple-choice exam in the classroom (long-term testing session), and after the exam (which had no time limit), they completed the STAI and PSQ index.

#### Training (day 1)

The training consisted of a history lesson about the ancient city of Petra and the Nabatean culture and commerce, which was presented by their teacher of Political Economics. As a tool to provide the lecture, she used a PowerPoint presentation, which was shown to the students in the classroom by using a projector. Each slide was presented for about 1.5 min as she commented on them orally.

#### Short-term testing session (day 1)

Immediately after training, students made a multiple-choice exam consisting of 13 questions with four possible answers to each question and one correct answer. The answers to all the exam questions were among the information written on the slides. Students had no time limit to solve the exam. Half of the students of each course received the same set of 13 questions, whereas the other half received a different set of questions.

#### Targeted Memory Reactivation (day 1)

Memory cueing during sleep was carried out on the first night of the experiment. The students received a dispenser that was programmed to spray the congruent (coconut) or incongruent (violet) odor every 15 min, depending on the group they were assigned to. Volunteers were instructed to activate the dispenser at the time they went to sleep on the night of day 1 and to set the finishing time 1.5 h after that. It is important to highlight that the participants did not know which odor they received.

#### Long-term testing (day 8)

One week later, in the classroom and without any odor, each subject had to make a multiple-choice exam different from the one that they had answered on day 1. Again, they had no time limit to solve the exam. In each classroom, half the students received the questions that their partners had answered the week before and vice-versa.

### Control measures

As this study was carried out during the COVID-19 pandemic, when the levels of anxiety in the population were increased and a worse quality of sleep was reported^30–32^, we controlled that both groups had a similar level in both variables. For that, we evaluated the STAI and PSQ index. The PSQ index assesses sleep quality during the last 4 weeks. We chose it because a poor sleep quality sustained in time could potentially affect memory acquisition and consolidation^2^. Thus, if students in the No-TMR group had significantly worse sleep quality than the TMR group, differences in performance could be attributed to poor sleep quality.

### Experimental groups

Subjects were randomly assigned to one of two conditions: the “TMR” and the “No-TMR” groups.

#### Targeted Memory Reactivation” group (TMR, n = 22)

Participants were trained on day 1 on the presence of coconut odor. They received the reactivation using the same odor cue during the first night of sleep and were then finally tested on day 8.

#### No-Targeted Memory Reactivation” group (No-TMR, n = 23)

Participants were trained on day 1. They received the reactivation session using a different odor (violets), as a control condition, during the first night of sleep and were then finally tested on day 8.

### Statistical analysis

Data were statistically analyzed with SPSS version 25 (IBM Corporation). We calculated the percentage of correct answers during the short-term and long-term testing. We also calculated the memory change for each subject as [(# of correct responses at the long-term testing session—# of correct responses at the short-term testing session)*100/ # of correct responses at the short-term testing session].

The percentage of correct answers at the short- and long-term testing sessions was analyzed with repeated measures ANOVA, with “group” as between-subjects factor with two levels (TMR and No-TMR) and “session” as within-subjects factor with two levels (short- and long-term testing). Furthermore, we compared the memory change between groups with a two-tailed t-test and performed two separate one-sample t-tests to compare the memory change of both groups to the value zero. Alpha was set at 0.05.

We also analyzed the STAI and PSQ index scores with one-way ANOVAs with “group” as between-subjects factor with two levels (TMR and No-TMR). It is important to highlight that three of the students of the No-TMR group did not complete the STAI on day 1, six did not complete the STAI on day 8, six did not complete the PSQ index, and six did not complete the Trait Anxiety Inventory, and that two of the students of the TMR group did not complete the STAI on day 8, three did not complete the PSQ index, and three did not complete the Trait Anxiety Inventory. We report partial eta squared (η^2^p) and Cohen’s d as effect size estimates.

## Data Availability

The raw data supporting the conclusions of this article can be found in: https://zenodo.org/record/6571022.
